# Supplementary material for: Trehalose Suppresses Lysosomal Anomalies in Supporting Cells of Oocytes and Maintains Female Fertility
Source: Nutrients. 2022 May 22;14(10):2156. doi: 10.3390/nu14102156 (PMC9148094; doi:10.3390/nu14102156)
Supplement: Supplementary file 1 [file nutrients-14-02156-s001.zip › nutrients-1700907-supplementary.pdf]

Supplementary Table S1

Number of patients	15
Age (years)	37.5 ± 1.0
Day 3 FSH (mIU/mL)	9.7 ± 0.9
Day 3 LH (mIU/mL)	3.7 ± 1.1
Day 3 estradiol (pg/mL)	60.1 ± 17.7
Day of hCG estradiol (pg/mL)	2,932.9 ± 387.3
Day of hCG progesterone (ng/mL)	1.1 ± 0.1
Day of hCG FSH (mIU/mL)	16.4 ± 1.0
Day of hCG LH (mIU/mL)	2.9 ± 0.8
Ampoules of gonadotropin used*	23.3 ± 2.0
Days of stimulation	13.9 ± 0.4
Number of follicles aspirated	17.1 ± 2.3
Number of oocytes retrieved	11.3 ± 1.5
Number of mature COCs**	4.5 ± 1.4
Number of immature COCs	4.3 ± 0.6
Number of dysmature COCs	2.4 ± 0.4
Percentage of embryos fertilized	65.5 ± 4.5

*1 ampoule = 75IU
**COCs: Cumulus-oocyte complex

Supplementary Table S1. Clinical demographic data of patients. Values are expressed as mean ± SE.
